# Chemical composition, antimicrobial and antibiofilm activity of the essential oil and methanol extract of the Mediterranean cypress (*Cupressus sempervirens* L.)

**DOI:** 10.1186/1472-6882-14-179

**Published:** 2014-06-02

**Authors:** Samy A Selim, Mohammed E Adam, Sherif M Hassan, Abdulrhman R Albalawi

**Affiliations:** 1 Department of Clinical Laboratory Sciences, College of Applied Medical Sciences, Aljouf University, P.O. 2014, Sakaka, Saudi Arabia; 2 Botany Department, Faculty of Science, Suez Canal University, P.O. 41522, Ismailia, Egypt; 3 West Kordufan University (WKU), Nuhud, Sudan; 4 Botany Department, Faculty of Science, Beni Suef University, Beni Suef, Egypt

**Keywords:** Mediterranean *Cupressus sempervirens* L, Methanol extract, Essential oil, GC-MS, Antimicrobial activity, Antibiofilm activity

## Abstract

**Background:**

*Cupressus sempervirens* is a medicinal plant traditional, its dried leaves are used in treatment of stomach pain, diabetes, inflammation, toothache, laryngitis and as contraceptive.

**Methods:**

The present study was conducted to evaluate the *in vitro* antimicrobial, antibiofilm and determination chemical contents of the essential oil (Eo) and methanol extract from Mediterranean *C. sempervirens* L. The chemical composition of a hydrodistilled Eo of *C. sempervirens* was analyzed by a GC and GC/MS system.

**Results:**

A total of 20 constituents representing 98.1% of the oil were identified: α-pinene (48.6%), δ-3-carene (22.1%), limonene (4.6%) and α-terpinolene (4.5%) were the main components comprising 79.8% of the oil. The antimicrobial test results showed that the methanol extract of *C. sempervirens* strongly inhibited the growth of the test bacteria studied, except for yeast species while the Eo had moderate antibacterial, but no anti-candida activity. *Klebsiella pneumoniae* was proven to be the most susceptible against methanol extract. The exposure time of Eo and methanol extract for complete inhibition of cell viability of *K. pneumoniae* was found to be 250 μg at 30 min and 500 μg at 120 min, respectively. The antibiofilm potential of the samples was evaluated using methods of PVC microtiter and eradication on biomaterial. Visual results showed visible biofilm eradication from the surface of intravenous infusion tube at 500 μg of Eo and methanol extract.

**Conclusions:**

The results presented here may suggest that the Eo and extracts of *C. sempervirens* possess antimicrobial and antibiofilm properties, and therefore, can be used as natural preservative ingredients in food and/or pharmaceuticals.

## Background

The genus *Cupressus* (Cupressaceae) consists of twelve species spread across North America, the Mediterranean Basin, and subtropical Asia at high altitudes [1]. *C. sempervirens* is a considered to be a medicinal tree, as its dried leaves are used for stomach pain, as well as to treat diabetes, and its dried fruit is used to treat inflammation, toothache, and laryngitis and as a contraceptive and astringent [2]. In addition, its dried seeds have been used to treat wounds, ulcers, bruises, sores, pimples, pustules, skin eruptions, and erysipelas, and the essential oil from the leaves and cones is used externally for headache, colds, cough, and bronchitis. With respect to these medicinal and pharmacological advantages, *C. sempervirens* is widely used as a cosmetic ingredient in perfumery and soap-making, including its essential oil distilled from shoots [3]. This species has many specific botanical features, including tolerance to drought, air currents, wind-driven dust, sleet, and atmospheric gases, a well-developed root system, the ability to flourish in both acidic and alkaline soils, and seeds that are easily collected for oil extraction. These seeds can be an industrial oil source and the extracted oil with a weak valued cost price might be used for different applications [4].

The geographic area of *Cupressus* genus is limited to the northern hemisphere and many species have been studied [5]. To the best of our knowledge, there are many papers report on the chemical composition of essential oils of *C. sempervirens* grown in north Mediterranean basin [6-8]. Few studies have investigated their antimicrobial activities [9-11]. The aim of this work was to assay the main constituent of the essential oil obtained from leaves of Mediterranean *C. sempervirens*, and to carry out a comparative evaluation of their antibacterial, antibiofilm activities and chemical contents.

## Methods

### Plant materials

The aerial parts of *C. sempervirens* L. were collected from the random gardens in Sakaka, Aljouf (Saudi Arabia) on August 2013. *C. sempervirens* is largely used as windbreaks and ornamentals throughout northern of Saudi Arabia, and used as traditional medicines to treat cough, influenza, and rheumatism by local people (information has been taken from native people). Specimens were identified at the Aljouf University (Aljouf, Saudi Arabia) and voucher specimen (No. 71) was deposited at the Herbarium of the Department in the cited university.

### Preparation of the methanol extract

The powder form of *C. sempervirens* (50 g) was extracted with methanol (200 ml X 3 times) at room temperature. The methanol extract was combined and evaporated by a vacuum rotary evaporator at 45°C to the dried powdered form (yield 2.6%, w/w). The resulting extract was then lyophilized and kept in the dark at +4°C until tested.

### Preparation of essential oil

Eo was obtained using the Clevenger hydrodistillation method. The plant material (about 300 g), was cut into small pieces, and placed in a flask (4 l) together with doubly distilled water (1.5 l). The mixture was boiled for 3 h, the collected Eo was dried with anhydrous sodium sulphate and kept at −18°C until use.

### Determination of total flavonoids

The aluminum chloride method was used for the determination of the total flavonoid content of the methanol extracts. Aliquots of extract solutions were taken and made up the volume 3 ml with methanol. Then 0.1 ml AlCl_3_ (10%), 0.1 ml Na-K tartarate and 2.8 ml distilled water were added sequentially. The test solution was vigorously shaken. Absorbance at 415 nm was recorded after 30 minutes of incubation. A standard calibration plot was generated at 415 nm using known concentrations of quercetin. The concentrations of flavonoid in the test samples were calculated from the calibration plot and expressed as mg quercetin equivalent/g of sample.

### GC and GC-MS analysis conditions of the essential oil

GC analysis was performed on a Hewlett Packard 5890 II gas chromatograph equipped with a FID and HP-5 ms capillary column (bonded and cross-linked 5%- phenyl-methylpolysiloxane 30 m · 0.25 mm i.d., film thickness 0.25 lm). Injector and detector temperatures were set at 220 and 290°C, respectively. The oven temperature was held at 50°C for 3 min, then programmed to 240°C at a rate of 3°C/min. Helium was the carrier gas, at a flow rate of 1 mL/min. Diluted samples (1/100 in acetone, v/v) of 1.0 lL were injected manually and in the splitless mode. Quantitative data were obtained electronically from FID area percent data. GC-MS analysis of the Eo was performed under the same conditions with GC (column, oven temperature, flow rate of the carrier gas) using a Hewlett Packard 5890 II gas chromatograph equipped with a mass selective detector in the electron impact mode (70 eV). Injector and MS transfer line temperatures were set at 220 and 290°C, respectively. The components were identified based on the comparison of their relative retention time and mass spectra with those of standards, NBS75K library data of the GC-MS system and literature data [12]. Alkanes were used as reference points in the calculation of relative retention indexes (RRI).

### Antimicrobial tests

#### Microbial strains

The bacterial and yeast strains used in this work are described in Table 1. These microbial strains were isolated from human and food beings and belong to the microbiological laboratory collection of the department of microbiology from Aljouf University, Saudi Arabia. Nutrient agar (for bacterial strains) and Sabouraud dextrose agar media (for yeast strains) were inoculated with this suspension of the respective organism and poured into a sterile petri dish.

**Table 1:** **Antimicrobial activity of the essential oil and methanol extract from *of *** **Mediterranean cypress *Cupressus sempervirens *** **L**

		**Essential oil**			**Methanol extract**		
	**Origin**	**DD**^**a**^	**MIC**	**MBC**	**DD**	**MIC**	**MBC**
**Gram positive bacteria**							
***Bacillus cereus***	**Food**	-	-	-	-	-	-
***Bacillus subtilis***	**Human**	-	-	-	-	-	-
***Enterococcus feacalis***	**Human**	-	-	-	4	250	250
***Serratia marcescens***	**Human**	-	-	-	-	-	-
***Staphlococcus aureus***	**Human**	7	250	250	6	125	125
**Gram negative bacteria**							
***Aeromonas hydrophila***	**Human**	-	-	-	-	-	-
***Escherichia coli***	**Human**	-	-	-	-	-	-
***Klebsiella pneumoniae***	**Human**	7	62.5	125	12	62.5	62.5
***Proteus vulgaris***	**Human**	-	-	-	-	-	-
***Pseudomonas aeruginosa***	**Human**	-	-	-	8	125	125
***Salmonella indica***	**Food**	4	250	250	4	125	125
**Yeast**							
***Candida albicans***	**Human**	-	-	-	-	-	-
***Saccharomyces cerevisiae***	**Human**	-	-	-	-	-	-

MIC (minimum inhibition concentration) and MBC (minimum bactericidal concentration) as μg/ml of essential oil or methanol extract; (−) no antimicrobial activity. Values are average of triplicate.

^a^Inhibition zone in diameter (mm) around the discs impregnated with essential oil and methanol extract (100 μg/disc).

#### Disc-diffusion assay

The agar diffusion assay was performed according to the modified Kirby-Bauer disc diffusion method [13]. One ml of each test organism liquid culture was individually suspended in 3 ml of a 0.9% NaCl solution. The Eo and methanol extract were dissolved in 10% dimethylsulfoxide (DMSO) to a final concentration of 30 mg/ml as stock solution and sterilized by filtration through 0.45 μm Millipore filters. Antimicrobial tests were then carried out using 100 μl of suspension containing 10^8^ cfu/ml of bacteria and 10^6^ cfu/ml of yeast spread on nutrient agar and Sabouraud dextrose agar media, respectively. The discs (6 mm in diameter) were impregnated with 100 μg of the essential oil and methanol extract, and then placed onto inoculated agar. Negative controls were prepared using the same solvent employed to dissolve the extract. The inoculated plates were incubated at 37°C for 24 h for clinical bacterial strains and 48 h for yeast isolates. Antimicrobial activity was evaluated by measuring the zone of inhibition against the test organisms.

#### Micro-well dilution assay of MIC and MBC

The minimal inhibitory concentration (MIC) values of the Eo and methanol extract were studied using the micro-well dilution method for the bacterial strains which were sensitive to Eo and methanol extract in the disc diffusion assay. In brief, the 96-well plates were prepared by dispensing 95 μl of nutrient broth and 5 μl of the inocula into each well. The inocula of the bacterial strains were prepared from 12 h broth cultures and suspensions were adjusted to 0.5 McFarland standard turbidity. One hundred μl aliquot from the stock solutions of the Eo and methanol extract initially prepared at the concentration of 250 μg/ml were added into the first wells. Then, 100 μl from their serial dilutions were transferred into six consecutive wells. The last well containing 195 μl of nutrient broth without the compound and 5 μl of the inocula on each strip was used as a negative control. The final volume in each well was 200 μl. The plate was covered with a sterile plate sealer. The contents of each well were mixed on a plate shaker at 300 rpm for 20s and then incubated at appropriate temperatures for 24 h. Microbial growth was determined by plating 5 μl samples from clear wells on nutrient agar medium. The extract tested in this study was screened twice against each organism. The MIC was defined as the lowest concentration of the compounds to inhibit the growth of microorganisms. MBC (minimum bactericidal concentration) is usually an extension from the MIC, where the organisms quantitatively indicate the minimum concentration when no viable organism appears in the culture [14].

#### Cell viability assay for *Klebsiella pneumoniae*

Each of the tubes containing bacterial suspension (approximately 10^6^ cfu/mL) of *Klebsiella pneumonia* was inoculated with 250 and 500 μg/ml of *C. sempervirens* Eo and methanol extract in 10 mL nutrient broth, and kept at 37°C. Samples for viable cell counts were taken at 0, 10, 20, 30, 60 and 120 min time intervals. The viable plate counts were monitored as followed: 100 μl sample of each treatment was diluted and spread on the surface of MHB agar. The colonies were counted after 24 h of incubation at 37°C. The controls were inoculated without Eo for each bacterial strain with the same experimental condition as mentioned above [15,16].

### Antibiofilm tests

#### Biofilm assay

The biofilm producing ability of *K. pneumoniae* was tested by staining the heat fixed cells with crystal violet. The slides were observed under the microscope. The biofilm was further assessed by using the crystal violet (CV) assay done in microtitre plate [17]. The procedure involved washing the plates after incubation, three times with sterile distilled water to remove loosely associated cells. The plates were air-dried and then oven-dried at 60°C for 45 min. Following drying, the wells were stained with 100 μL of 1% crystal violet and incubated at room temperature for 15 min after which the plates were washed 5 times with sterile distilled water to remove unabsorbed stain.

#### Study of the effect of Eo and methanol extract on biofilm formation by PVC microtiter

The effect of Eo and methanol extract on the formation of biofilm by *K. pneumoniae* was qualitatively estimated by a method described by Xiao et al. [18]. 40 μL of exponentially growing cells were dispensed in 96-well cell culture plates. Eo and methanol extract were added to the wells and incubated for 24 h at 37°C. The concentrations of extracts were ranged from 25 to 100 μg/mL. The medium without extracts was used as the non-treated control. It was observed that only Eo showed significant antibiofilm activity. After incubation, media and unattached cells were decanted and washed with Phosphate Buffer Saline (PBS). Then the plate was air dried and stained with 0.1% (v/w) Crystal Violet (Sigma-Aldrich, Germany).

#### Surface colonization and biofilm eradication on biomaterial

Fragments of intravenous infusion tube biomaterial (three pieces) were incubated with bacterial suspension in nutrient broth (NB) for 24 h at 37°C. The presence/reduction of biofilm biomass was evaluated by comparing the color intensity of biofilm deposit on biomaterials treated and untreated with Eo, methanol extract and ceftriaxone as reference antibiotic (500 μg/ml). The experiments were performed twice to confirm reproducibility of the results. Then, they were placed in 1 ml of sterile broth medium and the remaining bacterial deposit was removed by Phosphate Buffer Saline (PBS) for 5 min. Then the biomaterial was air dried and stained with 0.1% (w/v) Crystal Violet.

### Statistical analysis

The variations between experiments were estimated by standard deviations and the statistical significance of changes was estimated using the student’s t-test. Only the probability P ≤ 5% was regarded as indicative of statistical significance.

## Results and discussion

The natural products have been used as alternative medicines to conventional therapy and have gained interest in the researchers. This may be due to the perception that herbal products may be safe and have been used for many years as traditional medicines. Currently, researchers are focused on the therapeutic and pharmacological effects of natural products of plant origin.

### GC-MS analysis of essential oil and total flavonoids

The hydrodistillation of dried *C. sempervirens* gave browenish Eo (yield 2.6%, v/w). The identified compounds, qualitative and quantitative analytical results by GC and GC/MS are shown in Table 2. The GC–MS analysis of the Eo led to the identification of 20 different components, representing 98.1% of total oil constituents (Table 2). A total of 20 constituents representing 98.1% of the oil were identified; α-pinene (48.6%), δ-3-carene (22.1%), limonene (4.6%) and α-terpinolene (4.5%) were the main components comprising 79.8% of the oil. A portion (1.9%) of total composition was not identified. The Eo yield from the whole plants of *C. sempervirens* prepared by hydrodistillation method was 0.87% (v/w). GC/MS analysis revealed that the oil contain 20 components with α-pinene 48.6% of the oil.

**Table 2:** **Percentage chemical composition of the essential oil of Mediterranean cypress *Cupressus sempervirens *** **L.**

**Peak no**	**Compound**^**a**^	**KI**^**b**^	**Area**^**c **^**(%)**
1	Tricyclene	921	0.3
2	α-Thujene	922	1.4
3	α-Pinene	937	48.6
4	Camphene	948	0.5
5	Sabinene	974	2.0
6	β-Pinene	980	2.5
7	Myrcene	998	4.1
8	δ-3-carene	1012	22.1
9	p-Cymene	1021	0.2
10	Limonene	1024	4.6
11	γ -terpinene	1044	0.9
12	α-terpinolene	1064	4.5
13	Camphor	1104	0.1
14	Bronyl acetate	1289	0.2
15	Carvacrol	1304	0.1
16	β-Caryophyllene	1417	0.1
17	α-humulene	1559	0.3
18	Germacrene-D	1485	1.6
19	δ-cadinene	1527	0.5
20	α-cedrol	1610	3.5
**Total**			98.1
**Oil yield (%) (v/w)**			0.87

^a^Components were identified on KI and GC–MS (gas chromatograph coupled with mass spectrometry) and listed according to their elution on HP-5 MS capillary column (30 m).

^b^KI: Kovats indices on HP-5 MS capillary column in reference to C9-C28 n-alkanes [12].

^c^The 20 constituents identified represent 98.1% of total area.

The essential oils were obtained by steam-distillation from the leaves of *C. sempervirens* with yields (relative to dry weight material) of 0.87% (v/w). Algerian *C. sempervirens* had a yield at least three times inferior than the Cameroonian (1%) (v/w) [8]. The results are obtained by GC-MS analyses of the essential oils from *C. sempervirens* 20 compounds (98.1%) were identified; compared to Tunisian oil, they present 24 (92.95%) compounds identified [11]. The oils were predominantly composed of monoterpene hydrocarbons, with α-pinene as major constituent (48.6%). A similar result was obtained by Boukhris et al. [11] in their study of Tunisian *C. sempervirens*. They find that the largest group of constituents in the essential oil is the monoterpenes with α-pinene (37.14%). Similar result was found by Mazari et al. [9], when α-pinene is the major component of leaves essential oil, but it is presented in larger content (60.5%) compared with our study (48.6%). While, α-pinene is the second and the third major component, respectively, in the previous researches [11].

Flavonoids as one of the most diverse and widespread group of natural compounds are probably the most important natural phenols. These compounds possess a broad spectrum of chemical and biological activities including radical scavenging properties. Using the standard plot of quercetin (y = 0.0148×, R2 = 0.975) , the flavonoid contents of *C. sempervirens* leaves found ranging from 53 mg quercetin equivalent/g of dry sample.

### Antimicrobial activity

The *in vitro* antimicrobial potential of *C. sempervirens* Eo and methanol extract against a panel of microorganisms is shown in Table 1. Eo showed moderate *in vitro* antimicrobial activity against all tested bacteria, including Gram positive and Gram negative ones with diameter zones of inhibition 4 to 12 mm, along with MIC and MBC values ranging from 62.5 to 250 μg/ml. Whereas, the Eo showed less antimicrobial activity.

In the comparison of microbial sensitivity to both Eo and methanol extract, *K. pneumoniae* seem to be more sensitive than other infectious pathogens such as *Serratia marcescens*, *E. coli* and *Proteus vulgaris*. The results of antimicrobial activity are shown in Table 1. According to the statistical analysis, the gram-negative *K. pneumoniae* was the most sensitive strain, with MIC values ranging from 62.5 μg/ml. No remarkable activity was observed against the yeast that resulted the most resistant strain.

**Figure 1:**
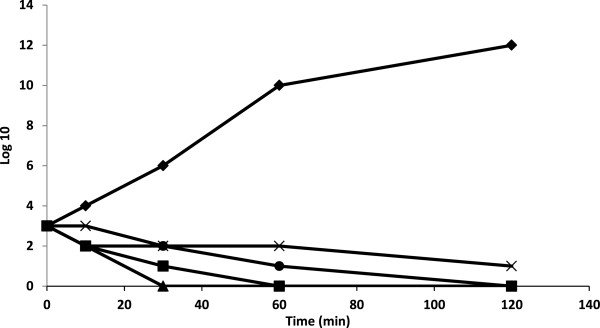
**Effect of essential oil and methanol extract from *of *Mediterranean cypress (*C. sempervirens *L.) as 0 (control, ♦), 250 μg/ml methanol extract (■), 500 μg/ml methanol extract (▲), 250 μg/ml Eo (x) and 500 μg/ml Eo ( )on viability of *K. pneumoniae.*** Control as no treatment with the essential oil and methanol extract. Values are the average of three individual replicates (means ± S.D). Differences between samples were determined by Student’s t-test and were considered to be significant when p ≤ 0.05 at least.

The antimicrobial activity of the *C. sempervirens* essential oils was more pronounced against Gram-positive than Gram-negative bacteria. This is a general observation derived from studies with essential oils from many other species [19,20]. Generally, the higher resistance among Gram-negative bacteria could be ascribed to the presence of their outer phospholipidic membrane, almost impermeable to lipophilic compounds [21]. The absence of this barrier in Gram-positive bacteria allows the direct contact of the essential oils hydrophobic constituents with the phospholipids bilayer of the cell membrane, where they bring about their effect, causing either an increase of ion permeability and leakage of vital intracellular constituents, or impairment of the bacteria enzyme [19]. The antimicrobial activity of the essential oils of *C. sempervirens* could, in part, be associated with theirs major constituents such as α- pinene and cedrol. These components have been reported to display antimicrobial effects [22,23]. The essential oils containing terpenes are also reported to possess antimicrobial activity [24], which are consistent with our present study. The antimicrobial compounds from plant source have increasing attention in recent years. Although there are many reports available on the antimicrobial properties of plants extracts, there are very few reports are available on the antibiofilm activities of plant extracts. Hence, the present study aimed to find the antibiofilm activities of different plant extracts. The antimicrobial activity of *C. sempervirens* Eo may be due the presence of phenolics, alkaloids, flavonoids, terpenoids and polyacetylenes. The antimicrobial activity of the plant extract is majorly attributed to the presence of phenolic compounds. Further, the effect on the cell viabilities of *K. pneumoniae* demonstrated that exposure of 250 and 500 μg/ml of *C. sempervirens* Eo and methanol extract had a potential antibacterial effect on the viabilities of *K. pneumoniae* strains. The exposure time of Eo and methanol extract for complete inhibition of cell viability of *K. pneumoniae* were found to be as 250 μg/ml at 30 min and 500 μg/ml at 120 min, respectively (Figure 1).

### Antibiofilm activity

**Figure 2:**
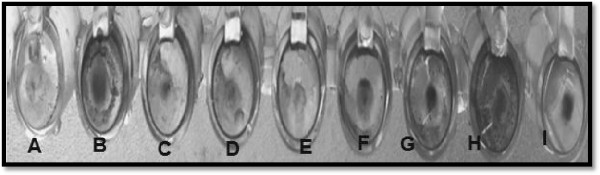
**Showed biofilm formation of *K. pneumoniae *in PVC microtiter plate wells arranged from low to high biofilm production.** Wells from left to right: **(A)** negative control (nutrient broth); **(B)** bacterial suspension supplemented by Eo 100 μg **(C)**, 50 μg **(D)**, 25 μg **(E)**; methanol extract 100 μg **(F)**, 50 μg **(G)**, 25 μg **(H)** and ceftriaxone as reference antibiotic 100 μg **(I)** of stained with 1% crystal violet solution after 24 h of incubation at 37°C.

The effect of Eo and methanol extract on the attachment of cells to polyvinyl chloride (PVC) was investigated. In the present study, out of the Eo and methanol extract used, Eo from *C. sempervirens* significantly shown antibiofilm activity. Growth of *K. pneumoniae* strain in PVC microtiter biofilm screening assay as described by Djordjevic et al. [17]. Generally, the use of Eo and methanol extract to inhibit *K. pneumoniae* attachment to PVC was successful with most extracts showing reduced biofilm biomass compared to the control biofilm (Figure 2).

**Figure 3:**
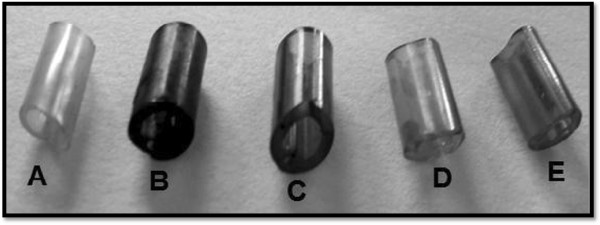
**Image of intravenous infusion tube colonized by *Klebsiella pneumoniae *** **biofilm: from left to right, (A) not colonized negative control, (B) treated positive control, (C) treated with 500 μg/ml methanol extract of *C. sempervirens*, (D) treated with 500 μg/ml Eo of *C. sempervirens *** **and (E) treated with 500 μg/ml ceftriaxone as reference antibiotic.**

However, none of the extracts was able to inhibit cell attachment completely, including the positive control, ceftriaxone. It was clearly indicated that there was tubes incubated with *C. sempervirens* showed reduction in EPS production at 100 μg *C. sempervirens* Eo and methanol extract. There is no report on antibiofilm activity of *C. sempervirens*. Among the medical biomaterials tested, intravenous infusion tube was the surface most prone to persistent colonization, since biofilms formed on it by *K. pneumoniae* was more easily to eradicate by the tested compounds. The obtained visual results showed visible biofilm eradication from the surface of intravenous infusion tube at 500 μg (Figure 3). Our study leads to the conclusion that Eo and methanol extract are able to efficiently reduce biofilms of *K. pneumoniae* on biomaterial surfaces.

The success of plant extracts in inhibiting cell attachment as shown in this study is a promising tool for reducing microbial colonisation on surfaces and epithelial mucosa which subsequently leads to infections. The ease with which the Eo and methanol extract inhibited cell attachment is confirmation of previous reports where it was found that inhibition of cell attachment to a substrate is easier to achieve than inhibiting the growth of an already established biofilm [25]. The success in inhibiting cell attachment can be explained in a number of ways. Firstly, cell attachment is the initial stage in biofilm formation following surface conditioning which creates a favourable environment for bacterial attachment [26]. During this stage however, most cells are still in the fluid phase and therefore still possess the same properties as planktonic bacteria making them susceptible to antimicrobial agents. In addition, surface conditioning is achieved by the adsorption of substances that include nutrients, organic and inorganic molecules, that are important for the growth of the cells, which in turn promotes cell adhesion. It can therefore be postulated that pretreatment of the surface with plant extracts produced an unfavorable film that repel the cells back into the fluid phase, thereby reducing surface adhesion. Some researchers have also demonstrated the success of coating medical devices with biocides such as silver to reduce microbial adhesion and the subsequent disease pathogenesis [27,28]. In contrast, some substances have been shown to play an important role in promoting microbial adhesion. These include albumin, gelatin, fibrinogen and casein [29]. The enhancement of cell attachment observed for some extracts in this study may be due to the presence of certain compounds within the extracts that provided a conditioning film promoting microbial adhesion. Also this enhancement effect confirms reports that some natural compounds promote the growth of microorganisms [30].

The enhanced attachment observed upon exposure to some extracts can therefore be postulated to be a result of the presence of compounds that favor the development of these biofilms. Further work involving analysis of the constituent compounds that promote biofilm growth need to be conducted to confirm this observation. It would be very interesting to investigate the type of phenolic compounds responsible for the antibiofilm activity of *C. sempervirens* Eo and this would be future scope of our study.

## Conclusion

As the industries tend to reduce the use of chemical preservatives in their products, EO of *C. sempervirens* with potential active antimicrobial properties might be considered as a natural source for the maintenance or extension of the shelf life of products. In addition, delectable taste of the EO at the concentrations needed for antimicrobial properties was a bonus to its antimicrobial effects. On the other hand, these EOs might also be considered for developing products for controlling microbial infections. These results indicate the possibility of exploitation of Eo and methanol extract originated from *C. sempervirens* as effective inhibitors of biofilm in *K. pneumoniae*. This is the first report on the antibiofilm activities of Eo and methanol extract originated from *C. sempervirens* in the literature. The isolated compounds are expected to be useful for the study of antibiofilm agents in the future. As these tests have all been done *in vitro*, the next step may be further investigations in animal models to see if infection can be inhibited by the EO.

## Competing interests

The authors declare no conflict of interests.

## Authors’ contributions

SAS, MEA, SMH and ARA participated in the design of the study data analyses and manuscript preparation. All authors read and approved the final manuscript.

## Pre-publication history

The pre-publication history for this paper can be accessed here:

http://www.biomedcentral.com/1472-6882/14/179/prepub

## References

[B1] Rawat P Khan MF Kumar M Tamarkar AK Srivastava AK Arya KR Maurya R Constituents from fruits of *Cupressus sempervirens* Fitoterapia 2010 81 162 166 10.1016/j.fitote.2009.08.014 19686818

[B2] Mascolo N Autore G Capasso F Menghini A Fasulo MP Biological screening of Italian medicinal plants for anti-inflammatory activity Phytother Res 1987 1 28 31 10.1002/ptr.2650010107

[B3] Usher GA Dictionary of plants used by Man 1974 London: Constable and Company

[B4] Nehdi IA *Cupressus sempervirens* var. Horizentalis seed oil: chemical composition, physicochemical characteristics, and utilizations Ind Crop Prod 2013 41 381 385

[B5] Pierre-Leandri C Fernandez X Lizzani-Cuvelier L Loiseau AM Fellous R Garnero J Andréoli C Chemical composition of cypress essential oils: volatile constituents of leaf from seven cultivated *Cupressus* species J Essent Oil Res 2003 15 242 247 10.1080/10412905.2003.9712130

[B6] Chanegriha N Baâliouamer A Meklati BY Favre-Bonvin J Alamercery S Chemical composition of Algerian cypress essential oil J Essent Oil Res 1993 5 671 674 10.1080/10412905.1993.9698304

[B7] Chanegriha N Baâliouamer A Meklati BY Chretien JR: Keravis G GC and GC/MS leaf oil analysis of four Algerian cypress species J Essent Oil Res 1997 9 555 559 10.1080/10412905.1997.9700776

[B8] Tapondjou AL Adler C Fontem DA Bouda H Reichmuthm C Bioactivities of cymol and essential oils of *Cupressus sempervirens* and *Eucalyptus saligna* against *Sitophilus zeamais* Motschulsky and *Tribolium confusum* du Val J Stored Prod Res 2005 41 91 102 10.1016/j.jspr.2004.01.004

[B9] Mazari K Bendimerad N Bekhechi C Fernandez X Chemical composition and antimicrobial activity of essential oils isolated from Algerian *Juniperus phoenicea* L. and *Cupressus sempervirens* L J Med Plants Res 2010 4 959 964

[B10] Emamia SA Asilia J Rahimizadehb M Fazly-Bazzazc BS Khayyat MH Chemical and antimicrobial studies of *Cupressus sempervirens* L. and *C. horizentalis* Mill. essential oils Iran J Pharm Sci 2006 2 103 108

[B11] Boukhris M Regane G Yangui T Sayadi S Bouaziz M Chemical composition and biological potential of essential oil from Tunisian *Cupressus sempervirens* L J Arid Land Stud 2013 22–1 329 332

[B12] Adams RP Quadrupole mass spectra of compounds listed in order of their retention time on DB-5 Identification of essential oils components by gas chromatography/quadrupole mass spectroscopy 2001 Stream, IL, USA: Allured Publishing Corporation, Carol 456

[B13] Selim SA Abdel Aziz MH Mashait MS Warrad MF Antibacterial activities, chemical constitutes and acute toxicity of Egyptian *Origanum majorana* L., *Peganum harmala* L. and *Salvia officinalis* L. essential oils Afr J Pharm Pharmacol 2013 7 725 735

[B14] Selim SA El Alfy S Al-Ruwaili M Abdo A Al Jaouni S Susceptibility of imipenem-resistant *Pseudomonas aeruginosa* to flavonoid glycosides of date palm (*Phoenix dactylifera* L.) tamar growing in Al Madinah, Saudi Arabia Afr J Biotechnol 2012 11 416 422

[B15] Selim SA Chemical composition, antioxidant and antimicrobial activity of the essential oil and methanol of the Egyptian lemongrass *Cymbopogon proximus* Stapf Grasas y Aceites 2011 62 55 61 10.3989/gya.033810

[B16] Selim SA Antimicrobial, antiplasmid and cytotoxicity potentials of marine algae *Halimeda opuntia* and *Sarconema filiforme* collected from Red Sea Coast World Acad Sci Eng Technol 2012 61 1154 1159

[B17] Djordjevic D Wiedmann M Mclands borough LA: microtitre plate assay for assessment of *Listeria monocytogenes* biofilm formation Appl Environ Microbiol 2002 68 2950 2958 10.1128/AEM.68.6.2950-2958.2002 12039754 PMC123944

[B18] Xiao J Zuo Y Liu Y Li J Hao Y Zhou X Effects of *Nidus Vespae* extract and chemical fractions on adherence and biofilm formation of *Streptococcus mutans* Arch Oral Biol 2007 52 869 875 10.1016/j.archoralbio.2007.02.009 17382894

[B19] Burt S Essential oils: their antibacterial properties and potential applications in foods-a review Int J Food Microbiol 2004 94 223 253 10.1016/j.ijfoodmicro.2004.03.022 15246235

[B20] Delamare APL Moschen-Pistorello IT Artico L Atti-Serafini L Echeverrigaray S Antibacterial activity of the essential oils of *Salvia officinalis* L. and *Salvia triloba* L. cultivated in South Brazil Food Chem 2007 100 603 608 10.1016/j.foodchem.2005.09.078

[B21] Selim S Hassan S Al Soumaa K EL Anzy S Prevalence, antibiotic resistance and *in vitro* activity of yogurt against some gram negative pathogenic bacteria isolated from Arar Hospital, KSA Life Sci J 2013 10 1450 1456

[B22] Demirci B Kosar M Demirci F Dinc M Baser KHC Antimicrobial and antioxidant activities of the essential oil of *Chaerophyllum libanoticum* Boiss. et Kotschy Food Chem 2007 105 1512 1517 10.1016/j.foodchem.2007.05.036

[B23] Yang JK Choi MS Seo WT Rinker DL Han SW Cheong GW Chemical composition and antimicrobial activity of *Chamaecyparis obtusa* leaf essential oil Fitoterapia 2007 78 149 152 10.1016/j.fitote.2006.09.026 17161919

[B24] Dorman HJD Deansm SG Antimicrobial agents from plants: antibacterial activity of plant volatile oils J Appl Microbiol 2000 88 308 316 10.1046/j.1365-2672.2000.00969.x 10736000

[B25] Cerca N Martins S Pier GB Oliveira R Azeredo J The relationship between inhibition of bacterial adhesion to a solid surface by sub-MICs of antibiotics and subsequent development of a biofilm Res Microbiol 2005 156 650 655 10.1016/j.resmic.2005.02.004 15950124 PMC1351067

[B26] Kumar CG Anand SK Significance of microbial biofilms in food industry: a review Int J Food Microbiol 1998 42 9 27 10.1016/S0168-1605(98)00060-9 9706794

[B27] Klueh I Wagner V Kelly S Johnson A Bryers JD Efficacy of silver- coated fabric to prevent bacterial colonization and subsequent device-based biofilm formation J Biomed Mater Res 2000 53 621 631 10.1002/1097-4636(2000)53:6<621::AID-JBM2>3.0.CO;2-Q 11074419

[B28] Hashimoto H Evaluation of the anti-biofilm effect of a new antibacterial silver citrate/lecithin coating in an *in vitro* experimental system using a modified robins device J Jpn Assoc Infect Dis 2001 75 678 685 10.11150/kansenshogakuzasshi1970.75.678 11558130

[B29] Meadows PS The attachment of bacteria to solid surfaces Arch Microbiol 1971 75 374 381 10.1007/BF00407699 4927242

[B30] Ofek I Hasty DL Sharon N Anti-adhesion therapy of bacterial diseases: prospects and problems FEMS Immunol Med Microbiol 2003 38 181 191 10.1016/S0928-8244(03)00228-1 14522453

